# Identification of kinases and regulatory proteins required for cell migration using a transfected cell-microarray system

**DOI:** 10.1186/s12863-015-0170-7

**Published:** 2015-02-05

**Authors:** Reiko Onuki-Nagasaki, Akira Nagasaki, Kazumi Hakamada, Taro QP Uyeda, Masato Miyake, Jun Miyake, Satoshi Fujita

**Affiliations:** Biomedical Research Institute, National Institute of Advanced Industrial Science and Technology (AIST), 1-1-1 Higashi, Tsukuba, Ibaraki 305-8566 Japan; Research Fellow of Japan Society for the Promotion of Science, 5-3-1 Kojimachi, Chiyoda-ku, Tokyo, 102-0083 Japan; Department of Mechanical Science and Bioengineering, Graduate School of Engineering Science, Osaka University, 1-3 Machikaneyama-cho, Toyonaka, Osaka 560-8531 Japan; Current address: Central Research Laboratories Sysmex Corporation, 4-4-4 Takatsukadai, Nishi-ku, Kobe 657-2271 Japan

## Abstract

**Background:**

Cell migration plays a major role in a variety of normal biological processes, and a detailed understanding of the associated mechanisms should lead to advances in the medical sciences in areas such as cancer therapy. Previously, we developed a simple chip, based on transfected-cell microarray (TCM) technology, for the identification of genes related to cell migration. In the present study, we used the TCM chip for high-throughput screening (HTS) of a kinome siRNA library to identify genes involved in the motility of highly invasive NBT-L2b cells.

**Results:**

We performed HTS using TCM coupled with a programmed image tracer to capture time-lapse fluorescence images of siRNA-transfected cells and calculated speeds of cell movement. This first screening allowed us to identify 52 genes. After quantitative PCR (qPCR) and a second screening by a conventional transfection method, we confirmed that 32 of these genes were associated with the migration of NBT-L2b cells. We investigated the subcellular localization of proteins and levels of expression of these 32 genes, and then we used our results and databases of protein-protein interactions (PPIs) to construct a hypothetic but comprehensive signal network for cell migration.

**Conclusions:**

The genes that we identified belonged to several functional categories, and our pathway analysis suggested that some of the encoded proteins functioned as the hubs of networks required for cell migration. Our signal pathways suggest that epidermal growth factor receptor (EGFR) is an upstream regulator in the network, while Src and GRB2 seem to represent nodes for control of respective the downstream proteins that are required to coordinate the many cellular events that are involved in migration. Our microarray appears to be a useful tool for the analysis of protein networks and signal pathways related to cancer metastasis.

**Electronic supplementary material:**

The online version of this article (doi:10.1186/s12863-015-0170-7) contains supplementary material, which is available to authorized users.

## Background

Cell migration is necessary for many physiological processes, such as tissue development, wound healing, and inflammation, as well as for pathological processes such as the metastasis of cancer cells [1]. Cell migration involves regulation of the cytoskeleton, substrate adhesion, membrane trafficking, and cell polarity. As a consequence, the regulation of cell migration is complex [2], requiring coordinated spatiotemporal control of such cellular events during the migration process. An understanding of the relationships of these phenomena to cell migration is important in the medical sciences, in particular, in areas such as cancer therapy. Wound-healing and Boyden chamber assays have been used in attempts to unravel the molecular mechanisms of cell migration, and they have provided information about some aspects of cell migration [3,4]. However, the molecular basis for the cooperativity between these events in migrating cells remains unclear. Thus, complete identification of all genes involved in cell migration is needed.

We developed a simple cell microarray chip for high-throughput phenotypic screening (HTS) that could be used to identify genes that are required for cell migration [5,6]. Our chip is based on the transfected cell microarray (TCM) technology [7]. Microarray spots, including plasmid DNA, siRNA, and transfection reagents, are printed on a glass slide that has been coated with type I collagen. Cells take up DNA and siRNAs from the spots, and the extent of their subsequent migration is measured. Our methodology has advantages over earlier methods, such as the wound-healing and Boyden chamber assays, in that (i) it can be used for efficient HTS of siRNAs and cDNAs and (ii) it excludes the possibility of contact inhibition and the release of inflammatory cytokines from wounded cells.

In a previous study, we performed a model screening using siRNAs directed against known motility-related genes to validate our methodology. In the present study, we preformed HTS and identified genes related to cell migration using our cell chip on which a kinome siRNA library had been spotted. Cell migration is a highly integrated and multistep process. Therefore, migration speeds should change when the function of any gene involved in any of the steps is impeded. We postulated that our approach would allow us to identify previously unrecognized genes involved in cell migration and to characterize the mechanisms that control cell migration.

## Results

### Screening for genes that regulate cell migration using TCM

Some kinases have been shown to be potential or even actual targets for anti-cancer drugs because such drugs function by altering signal transduction and changing the properties of cancer cells (http://www.cancer.gov/drugdictionary). Among available anti-cancer drugs directed against molecular targets, more than half target kinases. Therefore, we decided to search for kinases that regulate cell migration using a kinome siRNA library printed on a TCM chip. We used the commercially available Rat Kinase siRNA Set Version 1.0 from QIAGEN (Tokyo, Japan), which consists of siRNAs that target two different sites in each of 738 genes for kinases and related proteins without kinase activity, such as activators and inhibitors of kinases. On each glass slide, we printed four copies of ten different transfection mixtures, each containing siRNA as shown in Additional file 1. As in our earlier study, anti-paxillin (Pxn) and non-targeted siRNAs were printed on each slide as a positive and a negative control, respectively. To screen for genes required for cell migration, we produced 148 TCM chips with “4 × 12” grids (48 spots/chip). We used a line of NBT-L2b cells that is a subpopulation of cells isolated from parent NBT-II cells derived from a rat bladder carcinoma [8] because of their rapid (2 μm/min) and unidirectional movement on type I collagen-coated substrates [5]. When cells grow to a high density during experimental observations, it is hard to evaluate their migration on the small regions on a cell chip. The rapid migration of cells is, therefore, very important for successful screening on a cell chip because analysis can be completed before cell density becomes a problem. Suspensions of NBT-L2b cells were seeded onto the slides and incubated at 37°C (Additional file 1). After 24 h, fluorescent images of individual cells were captured for a 3-h period at 10-min intervals, and speeds of cell migration were calculated with Image J software and manual tracking plug-in (URL: http://rsb.info.nih.gov/ij/). We compared the mean speeds of individual cells that had been transfected with a target siRNA, a control siRNA [non-target (NT) siRNA or anti-Pxn siRNA] using the Mann-Whitney U-test. We identified 53 siRNAs, targeted to 52 genes, for which this comparison gave a P-value of less than 0.1 (Table 1 and Additional file 2).

**Table 1:** **Results of the first screening**

**Gene** ^**a**^	**Alias**	**Control**	**P value** ^**b**^
*Vapa*		Pxn	6.69E-13
*Prkd1*	*Prkcm*	Pxn	6.89E-09
*Kit*		Pxn	1.85E-07
*Btk*		Pxn	2.98E-07
*Srp72_siteA*		Pxn	1.61E-06
*Tfg*		Pxn	5.50E-06
*Akap12*	*AKAP12A*	Pxn	6.27E-06
*Pard3*		Pxn	1.02E-05
*Cpne3*		Pxn	1.12E-05
*Pank2*		Pxn	2.04E-05
*Mapk8ip*	*JIP1*	Pxn	6.26E-05
*Map4k4*		Pxn	2.00E-04
*Mapk8ip3*	*JSAP1*	Pxn	3.82E-04
*Irak1bp1*		Pxn	5.03E-04
*Riok3*		Pxn	7.00E-04
*Trib3*	*NIPK*	Pxn	1.40E-03
*Prkca*	*Pkca*	Pxn	2.60E-03
*Srpk1*		Pxn	2.90E-03
*Cdk13*	*Cdc2l5*	Pxn	4.00E-03
*Bmpr1a*		Pxn	4.00E-03
*Rapgef3*	*Epac*	Pxn	6.20E-03
*Srp72_siteB*		Pxn	6.40E-03
*Mark1*		Pxn	7.00E-03
*Stk24*		Pxn	7.17E-03
*Brdt*		Pxn	8.10E-03
*Lyn*		Pxn	8.70E-03
*Camk2b*	*Ck2b*	Pxn	8.90E-03
*Zap70*	*Srk*	Pxn	1.10E-02
*Camkv*	*1G5*	Pxn	1.20E-02
*Tgfbr1*		Pxn	1.80E-02
*Ksr1*	*Ksr*	Pxn	2.10E-02
*Tyrobp*	*Karap*	Pxn	2.40E-02
*Ibtk*		Pxn	2.70E-02
*Ripk1*		Pxn	3.20E-02
*Pik3c2a*		Pxn	4.10E-02
*Cerk*		Pxn	4.40E-02
*Chka*	*Chk*	Pxn	5.70E-02
*Pik3ca*		Pxn	8.90E-02
*Flt3*		Pxn	9.00E-02
*Mob3c*	*Mobkl2c*	Pxn	9.80E-02
*Dstyk*	*Ripk5*	NT	4.27E-06
*Map2k6*	*Mkk6*	NT	7.48E-06
*Dapk1*		NT	9.12E-06
*Prps2*		NT	1.85E-04
*Vrk3*		NT	6.84E-04
*Clk4*		NT	9.00E-04
*Ugp2*		NT	2.20E-02
*Cdc42bpb*		NT	5.05E-02
*Dgkg*	*Dagk3*	NT	6.50E-02
*Ckmt1b*	*Ckmt1*	NT	8.69E-02
*Nek4*		NT	9.97E-02
*Zfp512b* ^c^	*Urkl1*	NT	1.20E-02
*Ilk* ^c^		NT	8.30E-02

^a^The mean speeds of individual cells that expressed a test or control siRNA [non-target (NT) siRNA or anti-paxillin (Pxn) siRNA] were compared using the Mann-Whitney U-test (P < 0.1, control versus each siRNA). Fifty-one siRNAs, corresponding to 50 genes, had inhibitory effects on cell migration. ^b^The associated P-values are also shown. ^c^ The siRNAs that facilitated migration.

Of the 51 siRNAs that decreased the mean speed of migration of individual cells, as compared with the NT control siRNA, 40 siRNAs were more inhibitory with the anti-Pxn control siRNA. Two siRNAs (indicated *Ilk* and *Zfp512b* in Table 1) increased the speed of migration as compared to the effect of the NT siRNA.

### Second screening with a sensitive assay of motility

For a more accurate evaluation of cell migration in a second screening, we modified the screening method as follows. Fibronectin, one of the components of the transfection mixture, is essential for efficient transfection on cell chips, but NBT-II cells [9] and the derivative NBT-L2b cells [5] migrate more slowly on fibronectin than on collagen. Slower migration on a spot on the chip might, therefore, affect the accurate evaluation of cell speed. In addition, the fluorescence excitation required for visualization of cells might be toxic to migrating cells. Therefore, to evaluate the genes identified in the first screening in the absence of such potential confounding factors and for more accurate screening, we performed a second screening without exposure of cells to excitation light and, also, using a conventional method for transfection. After such transfection, cells were cultured for 38 h and transferred from 6-well microtiter plates to collagen-coated 24-well microtiter plates. Time-lapse imaging was initiated 6 h after the transfer of cells to the 24-well plates, because we had found that the migration speed is maximal at that time (data not shown). Knockdown of gene expression by siRNA is usually observed 24 to 48 h after transfection. After capturing a single fluorescent image for identification of transfected cells, we monitored cells by recording phase-contrast images at 5-min intervals for 6 h. In the second screening, we focused on the 51 siRNAs that had been found to have on inhibitory effect on cell migration plus the two siRNAs that accelerated migration.

**Figure 1:**
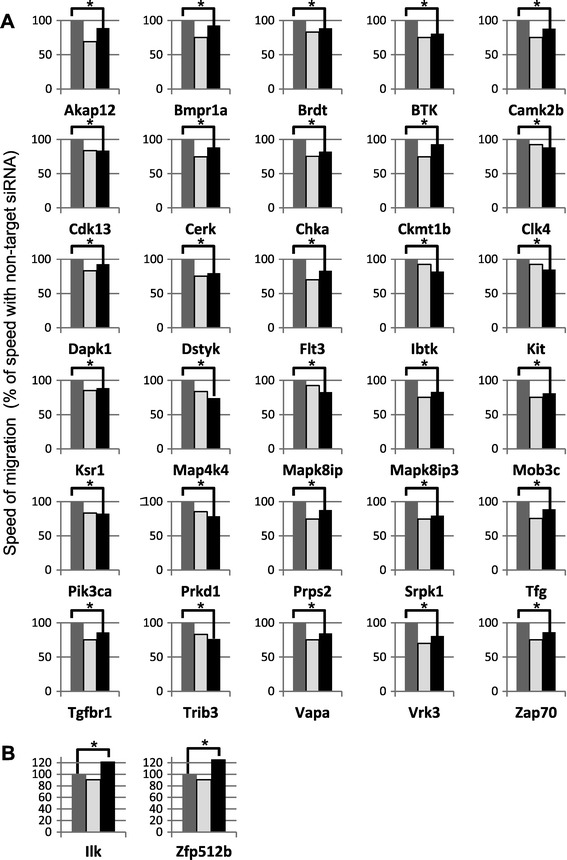
**The effects of siRNAs on cell migration.** The siRNAs identified in the first screening (black bars), non-target siRNA as a negative control (NT, dark gray bars), anti-paxillin siRNA as a positive control (Pxn, light gray bars) and the pVenus-N1 vector were introduced into NBT-L2b cells by conventional transfection. The migration speed of individual cells (N > 50) was analyzed by the ImageJ software with a manual tracking plug-in. The speeds of non-target siRNA-expressing cells and of cells expressing the other siRNAs were compared by the Mann-Whitney U-test (P < 0.05, NT *versus* each siRNA). siRNAs with inhibitory effects **(A)** and accelerating effects **(B)** on cell migration are shown. Positive and negative controls were measured together with 5 or 6 kinds of samples on one 24-well microplate in each experiment, therefore, the height of bars designated Pxn differs in the various histograms.

**Figure 2:**
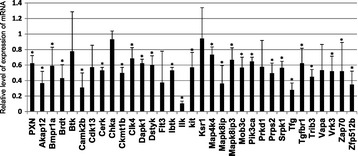
**siRNA-mediated knockdown of target mRNAs in NBT-L2b cells.** siRNAs were introduced into NBT-L2b cells with the pVenus-N1 vector. After 48-h incubation, total RNA was isolated and quantitative PCR (qPCR) was performed with the QuantiTect SYBR Green RT-PCR Kit (Qiagen). N = 4 replicates, average +/- SD *P < 0.05 in a comparison of control to each siRNA.

The second screening identified 30 siRNAs that targeted 30 genes and suppressed cell migration, with P-values below 0.05 by the Mann-Whitney U-test (Figure 1A). We also confirmed that two siRNAs, namely, anti-Ilk and anti-Zfp512b, had accelerative effects (Figure 1B). The knockdown efficacy of the active siRNAs, with the exception of the anti-Chka and anti-Ksr siRNAs, ranged from 20% to 70% (Figure 2). A statistical analysis of the effect of anti-Btk, anti-Cdk13, anti-Flt3, anti-Prkd1 and anti-Vapa siRNAs indicated that the knockdown effect was not statistically significant. The apparently positive results were caused, most likely, by low-level gene expression or weak amplification by PCR. Although the mean levels of expression (n = 4) of the *Chka* and *Ksr* genes were depressed to a relatively minor extent by the corresponding siRNAs, the inhibitory effects of these siRNAs on motility were highly reproducible. We decided, therefore, to use all the selected genes, including *Chka* and *Ksr*, in our subsequent analysis. In addition, we used anti-Pxn siRNA as a positive control, as described previously [5].

### Subcellular localization of products of the newly identified genes

**Figure 3:**
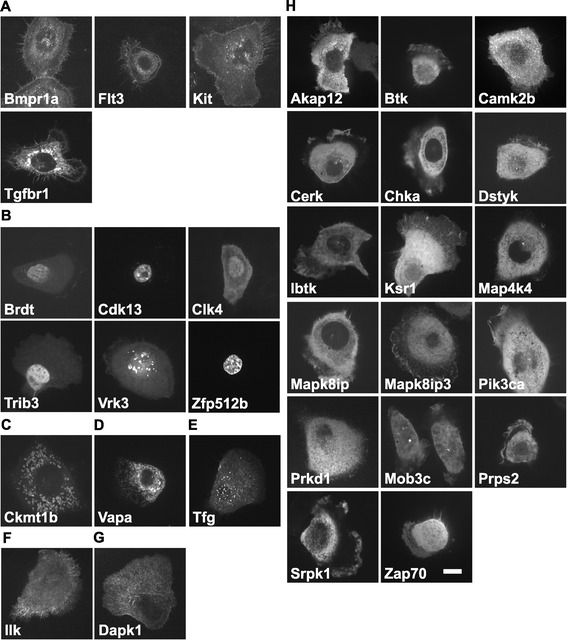
**Subcellular localization of target proteins in migrating cells.** Each expression vector was introduced into NBT-L2b cells by transfection on a collagen-coated glass dish. After 48 h, the localization of EGFP-fusion proteins was monitored by confocal microscopy. EGFP-fusion proteins were seen in the plasma membrane **(A)**, nucleus **(B)**, mitochondria **(C)**, ER **(D)**, transitional ER **(E)**, focal adhesions **(F)**, cytoskeleton **(G)** and lamellipodia and cytoplasm **(H)**. Bar represents 10 μm.

As described above, the 30 genes selected in the second screening had the apparent potential to accelerate and/or support the migration of NBT-L2b cells. In addition, the *Ilk* and *Zfp512b* genes had a potentially suppressive effect on cell migration. To investigate the localization of the proteins encoded by these genes in migrating NBT-L2b cells, we amplified the corresponding cDNAs from cDNA libraries. Each amplified full-length cDNA was cloned into the pEGFP vector for expression as a fusion protein with enhanced green fluorescent protein (EGFP). We failed to obtain full-length cDNAs for the *Btk*, *Camk2b*, *Flt3*, *Prkd1*, *Zfp512b*, and *Zap70* genes from cDNA libraries that had been prepared from NBT-L2b cells, presumably because of low levels of expression or incorrect DNA sequence information. However, we were able to amplify the six cDNAs from a human cDNA library. Expression plasmids encoding EGFP fusion proteins were introduced individually into NBT-L2b cells by transfection and transformants were placed in glass-bottomed dishes coated with type I collagen for observations by light microscopy. Twenty-four hours after transfection, we monitored the localization of each protein in NBT-L2b cells (Figure 3). As shown in Figure 3A, four receptor-type kinases, Bmpr1a [10], Flt3 [11], Kit [12], and Tgfbr1 [13], were localized on the plasma membrane, while Brdt [14], Cdk13 [15], Clk4 [16], Trib3 [17], Vrk3 [18], and Zfp512b [19] were detected in the nucleus (Figure 3B). Ckmt1 [20] and Vapa [21] were found in the mitochondria and on the endoplasmic reticulum (ER), respectively, as described previously (Figure 3C and D). Tfg was observed in transitional ER, as described previously (Figure 3E) [22]. Figure 3F shows that Ilk was localized to focal adhesion plaques, as reported previously [23], and Dapk1 was distributed in a fibrillar pattern, which suggested association with the cytoskeleton (Figure 3G) [24]. Other gene products were abundant in the cytoplasm, with noticeable concentration of some of them along the margins of lamellipodia (Figure 3H).

### Levels of expression of the identified genes in cells with high and low invasive capacity

**Figure 4:**
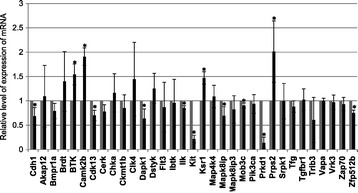
**Levels of expression of the identified genes in minimally invasive (NBT-T1) and highly invasive (NBT-L2b) cells.** Cells were cultured for 10 h on a collagen-coated dish. Total RNA was isolated and quantitative PCR (qPCR) performed with a QuantiTect SYBR Green RT-PCR Kit (Qiagen). Gray bars, NBT-T1; black bars, NBT-L2b *P < 0.05 in a comparison of NBT-T1 cells and NBT-L2b cells.

Nishi and coworkers isolated NBT-L2b cells, NBT-T1 cells and other cell lines from spontaneous variants of a cell line derived from a rat bladder tumor, NBT-II [8]. They examined the invasive potential of these lines using a Matrigel invasion chamber assay and they found that NBT-L2b cells had high invasive potential while NBT-T1 cells had low invasive potential. Furthermore, NBT-L2b cells migrated twice as fast as NBT-T1 cells on collagen-coated dishes (data not shown). Nishi and coworkers also reported that the level of expression of E-cadherin (cadherin 1, Cdh1) in NBT-L2b cells was lower than that in NBT-T1 cells, and that this difference might affect the speed of cell migration [8]. To examine whether the phenotypic difference between NBT-L2b and NBT-T1 cells reflects different levels of expression of the 32 selected genes, we performed qPCR (Figure 4). Expression of the *Cdh1* gene was used as a positive control. Levels of expression of the *Btk*, *Camk2b*, *Ksr1* and *Prps2* genes were somewhat higher (more than 1.4- to 2.0-fold difference) and those of the *Cdh1*, *Cdk13*, *Dapk1*, *Ilk*, *Kit*, *Map8ip*, *Mob3c*, *Prkd1* and *Zfp512b* genes were lower (from 0.1- to 0.9-fold difference) in NBT-L2b cells than in NBT-T1 cells.

### Pathway analysis

The signal transduction pathway that mediates cell migration is not well understood. Protein-protein interaction (PPI) databases are useful for analysis of the interactions between molecules and their functional roles in signaling networks. To determine the positions of the gene products of interest within networks, we tried to confirm the details of known signal-transduction pathways and then to identify novel pathways involved in cell migration, using the Genome Network Platform Viewer (URL: http://genomenetwork.nig.ac.jp/public/sys/gnppub/Top.do).

**Figure 5:**
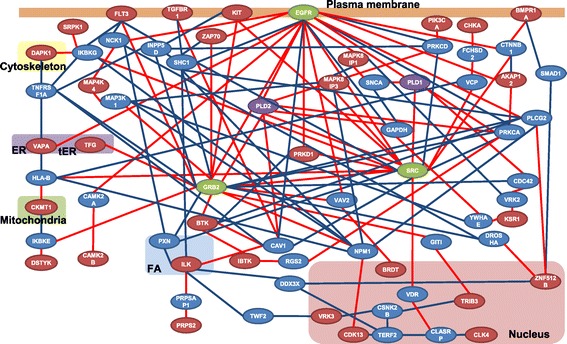
**Hypothetical network relationships and subcellular localizations of the products of genes identified by screening in the current study.** A protein-protein interaction network was constructed using the experimental data from this study and data sets provided by the Genome Network Platform Viewer. Red ovals, proteins identified by screening; blue ovals, proteins identified in PPI analysis with newly identified proteins and PLDs; green ovals, proteins functioning as apparent signaling hubs; purple ovals, PLD1 and PLD2; red lines, connections between PLDs and identified proteins; blue lines, connections between additional proteins, FA, Focal adhesions.

We tried first to draw maps of pathways among the proteins encoded by the newly identified genes, but sufficient information was not available to allow us to connect all the gene products on a single map. So we turned our attention to phospholipase D1 (PLD1) and phospholipase D2 (PLD2) because these enzymes interact with many binding partners and are known to serve as signaling hubs in cell migration [25]. We performed PPI analysis with either PLD1 or PLD2 and each of the identified kinases, hoping to generate a pathway that would link them together. Maps showing the signaling pathways between each PLD and individual proteins are shown in Additional files 3 and 4. We found that EGFR, GRB2, and Src appeared frequently in the PPI maps (orange circles, orange diamonds, and orange rounded squares in Additional files 3 and 4). Focusing on the pathways on the various maps, we tried to generate a unified pathway that would include the cellular location of each gene product. The red lines in Figure 5 show the connections between each identified protein (red circles) and PLDs (purple circles) that were obtained by PPI analysis (Additional files 3 and 4). Finally, we put all the proteins on this PPI map into the PPI database to identify additional connections among the proteins shown in Figure 5. These newly generated connections among proteins were added to the PPI map as blue lines (Figure 5). With 38 proteins newly added to the map (blue circles), we found that interactions among 30 of the 32 identified proteins (the exceptions being Cerk and Mob3c) were part of a single pathway (red circles) (Figure 5). No data for Cerk and Mob3c are currently available in the PPI database, and we were unable to place these two proteins on our map. The signaling pathways and subcellular localizations of the proteins on our map revealed that the epidermal growth factor receptor (EGFR) is the upstream regulator of the signal transduction network, while Src and GRB2 seem to represent nodes for control of respective downstream proteins required for the many cellular events involved in migration.

## Discussion

There are close to 40 anticancer drugs with specific molecular targets on the market today (URL: http://scads.jfcr.or.jp/db/table.html#table1). More than half of these drugs target kinases, and novel inhibitors of kinases might be valuable for the treatment of cancer in the future. Cell migration might be involved in cancer metastasis, but the molecular mechanisms that regulate cell migration are poorly understood. We developed a novel TCM technology as part of an effort to identify migration-related genes and analyze gene networks related to cell migration. In the first practical application of our method, we screened a kinome siRNA library in a search for genes that modulate cell migration.

We identified 53 potentially relevant siRNAs in our first screening of a commercially available siRNA library (Table 1 and Additional file 2). Two of these siRNAs, one targeting *Ilk* and the other targeting *Zfp512b*, actually enhanced cell migration (Table 1 and Additional file 2). Zfp512b is a zinc finger protein and ILK is an integrin-interacting protein kinase that has been identified as a potential PDK2 (PI 3-kinase-dependent kinase-2) since it is capable of phosphorylating PKB/Akt at Ser-473 and stimulating its activity [26].

In our second screening, we used a conventional transfection method and acquisition of phase-contrast images to exclude the possibility that fibronectin in the transfection mix might inhibit cell migration, and that continuous exposure to excitation light might be phototoxic to cells. We subjected the 53 candidate genes to the second screening (Figure 1) and confirmed the knockdown of expression of individual genes by qPCR (Figure 2). Among the initial 53 candidate genes, we identified 32 genes from inspection of the knockdown phenotype and level of expression. Thus, our TCM cell chip proved to be a powerful tool for bulk screening of genes. We performed qPCR four times to evaluate the knockdown efficacy for each siRNA, and we found that the *Chka-* and the *Ksr-*targeting siRNAs had only a minimal inhibitory effect on gene expression (Figure 2). Such suppression of expression of the *Chka* and *Ksr* genes was, however, observed in three and two replicates, respectively. Given that the expression of these genes in NBT-L2b cells might fluctuate with the status of cells, we included these two genes in our subsequent analysis. To our surprise, we also identified two siRNA that accelerated cell migration. These two siRNAs, targeting *Ilk* and *Zfp512b* (Additional file 2), were also confirmed by qPCR to knockdown their targets efficiently (Figure 2).

For convenience, we screened an existing kinome siRNA library to identify genes required for cell migration. To obtain more comprehensive information, we would need to use a whole-genome siRNA library, and the screening process would be very time-consuming. Therefore, we used signal network analysis to obtain information about connections that might have been missed in the screening of the kinome siRNA library.

To determine the cellular localization of the proteins encoded by the identified genes and to deduce their potential roles in migrating cells, we cloned the cDNAs of the 32 genes, as fusions with EGFP cDNA in plasmids and transfected NBT-L2b cells with the resultant plasmids. As noted above, six of the cDNAs were amplified from a human cDNA library. Because we were interested only in the localization of the products of the identified genes in migrating cells and because it is likely that the proteins are localized similarly in rat and human cells, we postulated that the use of clones from a human cDNA library would not invalidate our conclusions. The individual EGFP fusion proteins were found at characteristic locations in NBT-L2b cells that were migrating on collagen-coated dishes (Figure 3). Our results suggested that the products of each of the genes that we identified might be involved in a specific event as a component of a complex signaling-pathway network, as follows.

It has been reported that Chka, Kit, and Pik3ca function in PI3K/AKT signaling [27-30] and that Smad3, a transcription factor, is activated by Tgfbr1 and Trib3 [17,31]. Mapk8ip, Mapk8ip3 and Map4k4 participate in the regulation of JNK signaling [32-34]. JNK is involved in reorganization of the cytoskeleton via phosphorylation of Spir, DCX, and other microtubule-associated proteins [35]. Seven of the proteins, namely Akap12, Bmpr1a, Chka, Ksr1, Trib3, Vrk3, and Zap70, are involved in the ERK signaling pathway [18,30,36-40]. ERK1/2 is activated through the Ras-Raf-MEK1/2 cascade via the binding of growth factors to cognate-receptor tyrosine kinases at the plasma membrane. Activated ERK can be translocated to the nucleus to regulate transcription or it can be retained within the cytoplasm to regulate a variety of activities, which include cell motility, changes in organelle structure, integrin signaling, and cytoskeletal dynamics. For example, it has been proposed that phosphorylation of paxillin, FAK, calpain, and MLCK by ERK might regulate the dynamics of focal adhesion and membrane protrusion, thereby influencing cell migration [35]. Genes involved in ERK signaling form the largest group within the 32 genes that we identified.

We measured the levels of expression of the identified genes in highly-invasive (NBT-L2b) and minimally-invasive (NBT-T1) cells to determine whether levels of expression were correlated with invasive activity (Figure 4). Levels of expression of *Btk*, *Camk2b*, *Ksr1*, and *Prps2* were somewhat higher than those of the other genes. Expression of these four genes might be regulated at the transcriptional level, but this hypothesis needs to be validated. Levels of expression of *Cdh1*, *Cdk13*, *Dapk1*, *Ilk, Kit*, *Mapk8ip, Mob3c, Prkd1*, and *Zfp512b* were lower in NBT-L2b cells than in NBT-T1 cells (Figure 4). Expression of many of the genes might not be significantly higher in NBT-L2b cells than in NBT-T1 cells, if post-transcriptional regulation, for example by phosphorylation or genetic mutation, influenced the activity or activation of gene products rather than the level of gene expression.

We attempted to construct a signaling pathway using the data obtained from our second screening in order to enhance our understanding of the regulation of cell migration. However, since we had only screened genes for kinases and related proteins and also since the available PPI data were limited, we were unable to generate a useful map. Therefore, we chose PLDs as probes in an effort to identify the PPI network that involved the 32 proteins. The gene for PLD was identified as a migration gene in *Dictyostelium* cells and it is required for the migration of mammalian cells [41,42]. PLDs catalyze the hydrolysis of a terminal diester bond in phosphatidylcholine, which is abundant in the cell membrane, generating phosphatidic acid (PA) and diacylglycerol [43]. The PA that is produced by PLDs is an intracellular lipid mediator of many biological functions and has been found to be associated with numerous target proteins, such as Raf1, PI4P5 kinase, and mTOR [44]. Furthermore, PLD1 and PLD2 are known to bind to numerous proteins in cells and to form large signaling hubs [25]. The shortest pathways between PLDs and the identified proteins (with the exception of Cerk and Mob3c, for which PPI information was unavailable in the database) are shown in Additional files 3 and 4. Three proteins in particular, namely EGFR, GRB2 and Src, appear frequently on the PPI maps.

The interactions of 30 proteins were detected in one network when 38 proteins were newly included, with EGFR as the furthest upstream component of the pathway. EGFR is a receptor tyrosine kinase that is overexpressed in a variety of human epithelial malignancies, such as carcinomas of the lung, colon, ovary, bladder and head and neck [45]. Moreover, enhanced expression of EGFR and the subsequent increase in ERK and AKT signaling have been implicated in the progression of prostate cancer [46]. Furthermore, impairment of endocytic down-regulation of the activity of EGFR appears to contribute to the oncogenic phenotype [47,48]. Thus, EGFR-ERK signaling might be a major participant in the regulation of cell migration. Furthermore, the frequent appearance of Grb2 and Src during our PPI analysis indicated that these proteins might also play a role as signal-transduction hubs to connect the signal, via EGFR, from extracellular stimulation to key proteins in various cellular events.

Cell migration is controlled by several intracellular phenomena, which include membrane trafficking and the endocytosis of EGFR [49,50]. Ligand-induced endocytosis of EGFR has been reported to occur via two pathways, namely, clathrin-dependent endocytosis and caveolae-dependent endocytosis [49]. Although the kinases that we identified do not play any known role in the endocytosis of EGFR, the product of *Cav1*, a gene newly identified by PPI analysis, is a major participant in caveolae-dependent endocytosis [51]. It is possible that disruption of caveolae-dependent endocytosis of EGFR regulates the migration of NBT-L2b cell. Some of the proteins that we identified, such as Btk, Camk2b, Kit, Map8ip, Mapk8ip3, Prkd1 and Vapa, appear to regulate vesicular traffic according to the gene ontology (URL: http://www.ncbi.nlm.nih.gov) and might contribute to control of levels of expression of membrane proteins.

Six of the genes that we identified, namely, *BTK*, *Chka*, *Flt3*, *Kit*, *Pik3ca*, and *Tgfbr1* are the targets of anti-cancer drugs in current clinical trials (http://www.cancer.gov/drugdictionary). *Ksr1*, which is moderately overexpressed in highly invasive cells (Figure 4), activates ERK signaling and is a target of the Nm23-H1 suppressor of metastasis [52]. Therefore, *Ksr1* might be a candidate target for treatment of malignant cancers. Other proteins on our map might also be confirmed in future analysis to be candidate targets for anti-cancer drugs.

## Conclusions

Our selection method appears to provide a useful strategy for approaching the details of cell migration. The EGFR signaling pathway might be a major regulatory pathway in cell migration. Cell migration can be disrupted by knocking down individual genes, and such genes might become candidate targets for anticancer drugs.

## Methods

### Materials

Fibronectin was purchased from Life Laboratory Company (Yamagata, Japan). Rhodamine-labeled fibronectin was generated with an EZ-Label™ Rhodamine Protein Labeling Kit (Pierce Biotechnology, Rockford, IL, USA) according to the manufacturer’s protocol. Type B gelatin from bovine skin was purchased from Sigma (St. Louis, MO, USA). The pEGFP expression vector (BD Biosciences Clontech, Tokyo, Japan) and the pVenus-N1 expression vector (a gift from Dr. A. Miyawaki, RIKEN, Saitama, Japan) were used as reporter plasmids. Non-targeted (NT) siRNA and GFP-specific siRNA were obtained from QIAGEN (Tokyo, Japan; GFP, green fluorescent protein). Paxillin-specific (Pxn) siRNA (Rn_LOC360820_2_HP), which suppresses the rat *paxillin* gene was obtained from Qiagen. Since Pxn is a component of the adhesion complex and is required for cell migration [53], we chose expression of paxillin as a positive control in our screening system. Non-coated glass slides (Matsunami Glass Ind., Osaka, Japan) were used in this study. Rat Kinase siRNA Set Version 1.0 (Qiagen) was used as the kinome siRNA library.

### Culture and transfection of cells

NBT-L2b and NBT-T1 cells were obtained from the RIKEN Cell Bank (Tsukuba, Ibaraki, Japan) and grown in MEM (Sigma, Tokyo, Japan), supplemented with 10% fetal bovine serum (MP BioChemicals, Cleveland, OH, USA), non-essential amino acids (GIBCO-BRL, Gaithersburg, MD), sodium pyruvate (GIBCO-BRL), and a mixture of antibiotics, including penicillin and streptomycin (GIBCO-BRL). HiPerFect Transfection Reagent (Qiagen) was used for standard transfections according to the manufacturer’s protocol.

### Quantitative PCR (qPCR)

For isolation of total RNA, Isogen (Nippon Gene, Tokyo, Japan) was added to siRNA-transfected cells, non-transfected NBT-L2b cells, and NBT-T1 cells. qPCR was performed with the QuantiTect® SYBR® Green RT-PCR Kit (Qiagen) according to the manufacturer’s protocol. The primers for qPCR (Qiagen) are shown in Additional file 5.

### Gene screening by TCM (first screening)

The basic preparation of the transfection microarray was described in our previous reports [5,6]. In brief, 1 μg of pEGFP-N1 vector and an siRNA (20 μM) were suspended in serum-free Dulbecco’s Modified Eagle’s Medium (GIBCO-BRL). After addition of Lipofectamine™ 2000 (Invitrogen, Tokyo, Japan), the mixture was incubated at room temperature for 30 min. Rhodamine-labeled fibronectin (4 mg/ml) and 0.1% gelatin were added, and the resultant mixture was printed on collagen-coated glass slides with a microarray printer (KCS-mini; KUBOTA Comps., Osaka, Japan). Then, 2 × 10^5^ cells were seeded onto the printed glass slide and allowed to proliferate for 24 h. Fluorescence time-lapse images were recorded for 3 h at 10-min intervals with a Programmable Cellular Image Tracer (Olympus, Tokyo, Japan). The speed of migration of individual cells was analyzed with CellVoyager (Yokogawa, Tokyo, Japan) and ImageJ software with a manual tracking plug-in. The mean speeds of individual cells that expressed a test or control siRNA (NT siRNA or anti-Pxn siRNA) were used for statistical comparisons by the Mann-Whitney U-test (statistical significance recognized at P < 0.1, control *versus* each siRNA).

### Screening genes by a motility assay (second screening)

siRNAs from the first screening, NT siRNA, or anti-Pxn siRNA plus the pVenus-N1 vector were introduced into NBT-L2b cells by the conventional transfection method described above. After 38 h, siRNA-transfected cells (3 × 10^4^ cells) were diluted in 1 ml of DMEM/F12 Ham’s medium (Sigma), supplemented with 10% fetal bovine serum and a mixture of antibiotics, and seeded in wells of a non-treated 24-well microplate (Asahi Glass., Ltd, Tokyo, Japan) that had been coated with a 0.001% solution (w/v) of type I collagen (Research Institute for Functional Peptides, Yamagata, Japan). After a 3-h incubation, the medium was replaced with 1 ml of fresh DMEM/F12 Ham’s medium. The microplate was placed in a Programmable Cellular Image Tracer and incubated for at least 3 h. Fluorescence and phase-contrast snapshot images were captured and phase-contrast time-lapse images were recorded for 6 h at 5-min intervals with the Image Tracer, and analyzed with the ImageJ software. The migration speeds of cells that had been transfected with NT siRNA and the test siRNAs were compared by the Mann-Whitney U-test (statistical significance recognized at P < 0.05, NT *versus* each siRNA).

### Subcellular localization of gene products

Full-length cDNAs of genes identified in the second screening were cloned from cDNA libraries derived from NBT-L2b cells or from the human HeLa and HL60 cell lines. Details of expression vectors and cloned genes are shown in Additional file 6. The expression vectors were introduced into NBT-L2b cells with HiPerFect according to the manufacturer’s protocol. Transfected cells were incubated for at least 24 h and observed with a confocal laser scanning microscope (Yokogawa, Tokyo, Japan).

### Pathway analysis

We used PPI network data from the Genome Network Platform database (GNP, http://genomenetwork.nig.ac.jp/public/sys/gnppub/Top.do) to map the signaling pathways that connected the proteins that we had identified as migration-related. We entered the identified proteins and PLDs into the Path Search of the PPI database. From maps of signal pathways for each PLD and an identified protein, we generated integrated signal pathways. In addition, the intracellular localization of the identified proteins was added to the final map.

## Additional files

Additional file 1:
**Schematic representation of a TCM-based cell chip for monitoring cell migration.** The transfection mixture, consisting of Lipofectamine™ 2000, the pEGFP-N1 expression vector, siRNA, rhodamine-labeled fibronectin, and gelatin, was printed as a 4 × 12 grid on the surface of a collagen-coated glass slide by a high-precision inkjet microarrayer (KUBOTA Comps., Osaka, Japan). Red signals from rhodamine-labeled fibronectin identified the spots of the transfection mixture on the slide. For analysis, NBT-L2b cells were seeded and incubated on the printed chip for 24 h. Cells transfected with siRNA were recognized by the fluorescence of EGFP. Additional file 2:
**Effects of siRNAs in the first screening.** NBT-L2b cells were seeded onto a printed glass slide and fluorescence time-lapse images were recorded for 3 h at 10-min intervals with a Programmable Cellular Image Tracer (OLYMPUS, Tokyo, Japan), and analyzed by ImageJ software with a manual tracking plug-in. siRNAs are indicated by their target genes. Dark gray bars, non-target siRNA-transfected cells; light gray bars, anti-paxillin siRNA-transfected cells; black bars, cells transfected with specific siRNAs. ^**^P < 0.05 in a comparison of control to each siRNA. ^*^P < 0.1 in a comparison of control to each siRNA. Additional file 3:
**Protein-protein interactions for products of newly identified genes and PLD1.** Red squares, PLD1 and products of newly identified genes; orange circles, EGFR; orange rounded squares, Src; and orange diamonds, GRB2. Additional file 4:
**Protein-protein interactions for products of newly identified genes and PLD2.** Red squares, PLD2 and products of newly identified genes; orange circles, EGFR; orange rounded squares, Src; and orange diamonds, GRB2. Additional file 5:
**siRNAs identified in the second screening, their target genes, and the corresponding primers for qPCR.**
Additional file 6:
**List of cloned genes and their expression vectors.** All cDNA fragments were cloned into the multiple cloning sites of pEGFP-N1 or vectors in the pEGFP-C series.
