# Supplementary material for: Identification of kinases and regulatory proteins required for cell migration using a transfected cell-microarray system
Source: BMC Genet. 2015 Feb 5;16:9. doi: 10.1186/s12863-015-0170-7 (PMC4365556; doi:10.1186/s12863-015-0170-7)
Supplement: Additional file 1: — Schematic representation of a TCM-based cell chip for monitoring cell migration. The transfection mixture, consisting of Lipofectamine™ 2000, the pEGFP-N1 expression vector, siRNA, rhodamine-labeled fibronectin, and gelatin, was printed as a 4 × 12 grid on the surface of a collagen-coated glass slide by a high-precision inkjet microarrayer (KUBOTA Comps., Osaka, Japan). Red signals from rhodamine-labeled fibronectin identified the spots of the transfection mixture on the slide. For analysis, NBT-L2b cells were seeded and incubated on the printed chip for 24 h. Cells transfected with siRNA were recognized by the fluorescence of EGFP. [file 12863_2015_170_MOESM1_ESM.pdf]

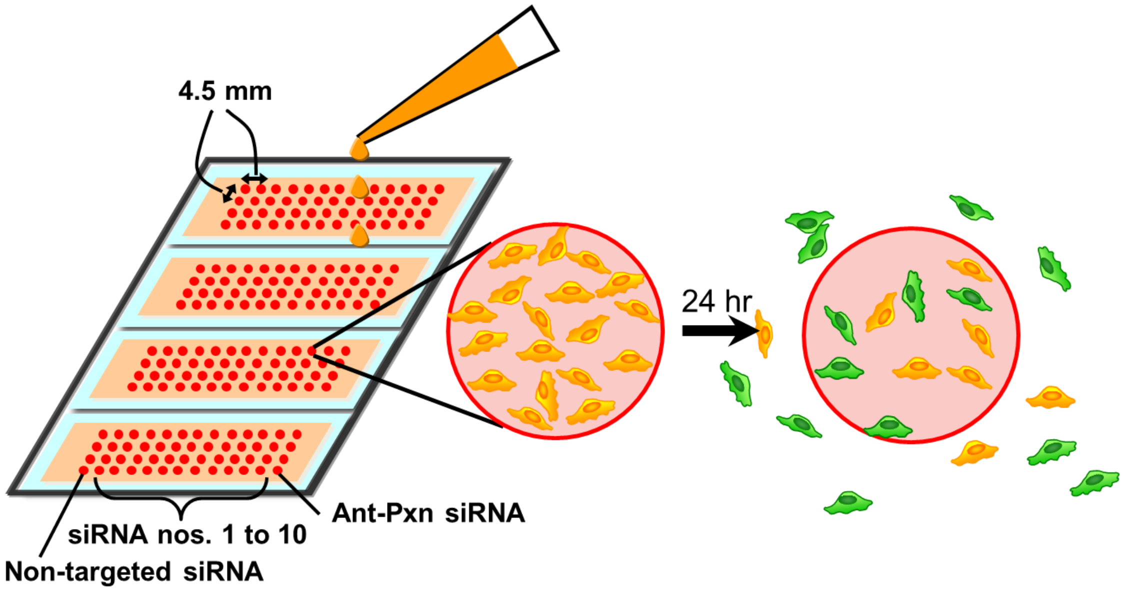

Additional file 1. Schematic representation of a TCM-based cell chip for monitoring cell migration.

The transfection mixture, consisting of Lipofectamine™ 2000, the pEGFP-N1 expression vector, siRNA, rhodamine-labeled fibronectin, and gelatin, was printed as a 4 x 12 grid on the surface of a collagen-coated glass slide by a high-precision inkjet microarrayer (KUBOTA Comps., Osaka, Japan). Red signals from rhodamine-labeled fibronectin identified the spots of the transfection mixture on the slide. For analysis, NBT-L2b cells were seeded and incubated on the printed chip for 24 h. Cells transfected with siRNA were recognized by the fluorescence of EGFP.
