# Supplementary material for: Identification of kinases and regulatory proteins required for cell migration using a transfected cell-microarray system
Source: BMC Genet. 2015 Feb 5;16:9. doi: 10.1186/s12863-015-0170-7 (PMC4365556; doi:10.1186/s12863-015-0170-7)
Supplement: Additional file 2: — Effects of siRNAs in the first screening. NBT-L2b cells were seeded onto a printed glass slide and fluorescence time-lapse images were recorded for 3 h at 10-min intervals with a Programmable Cellular Image Tracer (OLYMPUS, Tokyo, Japan), and analyzed by ImageJ software with a manual tracking plug-in. siRNAs are indicated by their target genes. Dark gray bars, non-target siRNA-transfected cells; light gray bars, anti-paxillin siRNA-transfected cells; black bars, cells transfected with specific siRNAs. **P < 0.05 in a comparison of control to each siRNA. *P < 0.1 in a comparison of control to each siRNA. [file 12863_2015_170_MOESM2_ESM.pdf]

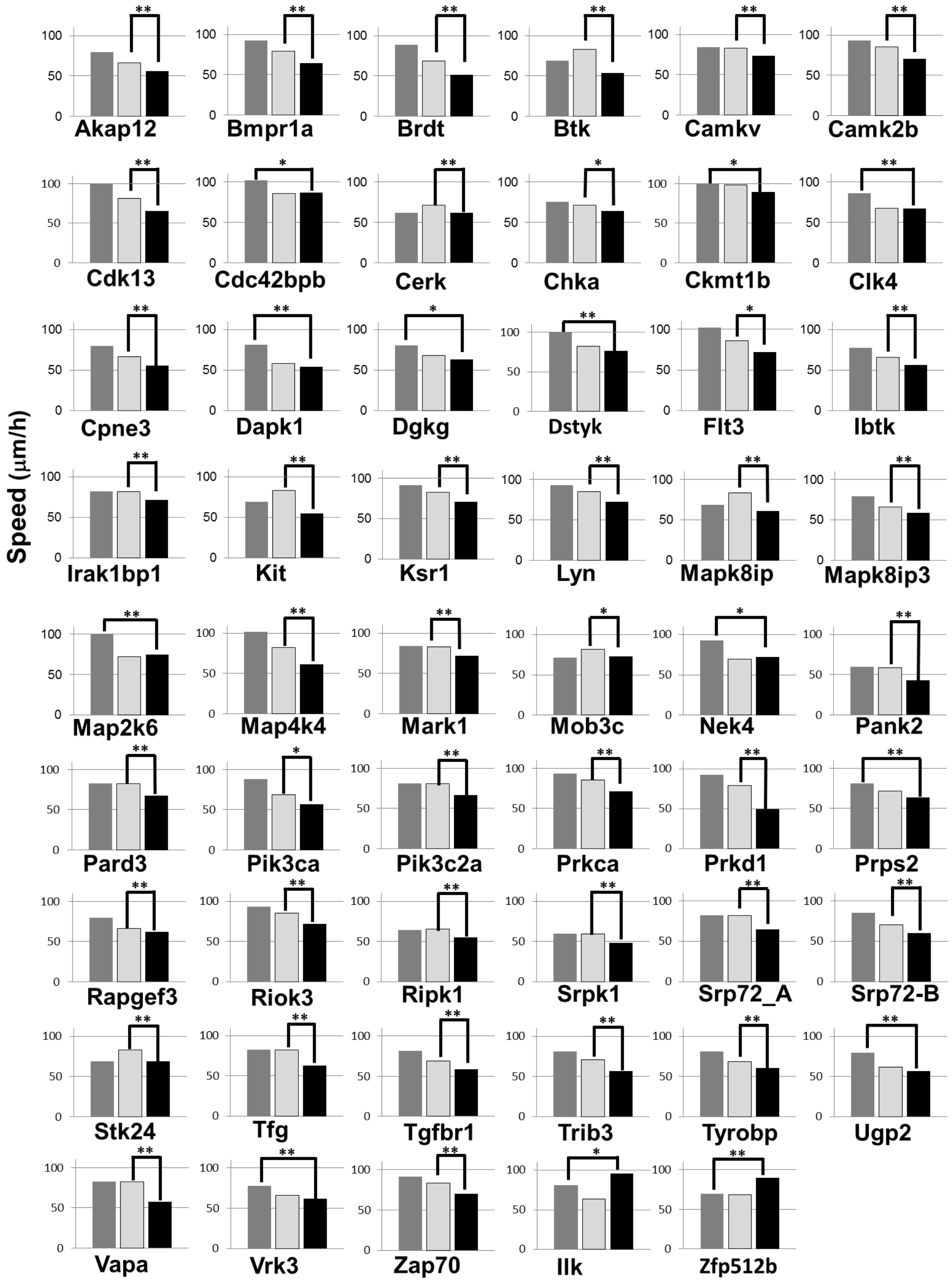

Additional file 2. Effects of siRNAs in the first screening.

NBT-L2b cells were seeded onto a printed glass slide and fluorescence time-lapse images were recorded for 3 h at 10-min intervals with a Programmable Cellular Image

Tracer (OLYMPUS, Tokyo, Japan), and analyzed by ImageJ software with a manual tracking plug-in. siRNAs are indicated by their target genes. Dark gray bars, non-target siRNA-transfected cells; light gray bars, anti-paxillin siRNA-transfected cells; black bars, cells transfected with specific siRNAs. $^{**}P<0.05$ in a comparison of control to each siRNA. $^{*}P<0.1$ in a comparison of control to each siRNA.
