# Supplementary material for: Identification of kinases and regulatory proteins required for cell migration using a transfected cell-microarray system
Source: BMC Genet. 2015 Feb 5;16:9. doi: 10.1186/s12863-015-0170-7 (PMC4365556; doi:10.1186/s12863-015-0170-7)
Supplement: Additional file 4: — Protein-protein interactions for products of newly identified genes and PLD2. Red squares, PLD2 and products of newly identified genes; orange circles, EGFR; orange rounded squares, Src; and orange diamonds, GRB2. [file 12863_2015_170_MOESM4_ESM.pdf]

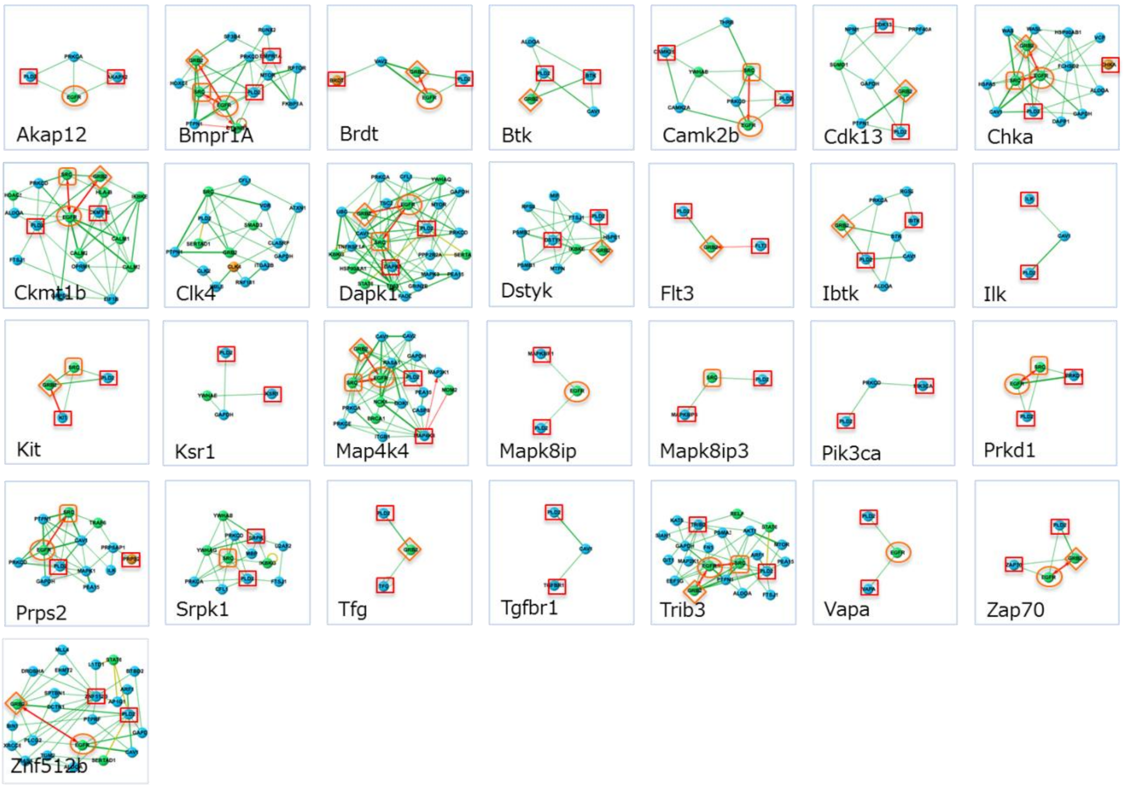

Additional file 4. Protein-protein interactions for products of newly identified genes and PLD2.

Red squares, PLD2 and products of newly identified genes; orange circles, EGFR; orange rounded squares, Src; and orange diamonds, GRB2.
