# Supplementary material for: Identification of kinases and regulatory proteins required for cell migration using a transfected cell-microarray system
Source: BMC Genet. 2015 Feb 5;16:9. doi: 10.1186/s12863-015-0170-7 (PMC4365556; doi:10.1186/s12863-015-0170-7)
Supplement: Additional file 5: — siRNAs identified in the second screening, their target genes, and the corresponding primers for qPCR. [file 12863_2015_170_MOESM5_ESM.pdf]

	Gene name	siRNA	qPCR primer
1	*Akap12*	Rn_Akap12_1 FlexiTube siRNA	Rn_Akap12_1_SG QuantiTect Primer Assay
2	*Bmpr1a*	Rn_Bmpr1a_4 FlexiTube siRNA	Rn_Bmpr1a_1_SG QuantiTect Primer Assay
3	*Brdt*	End of sale	Rn_Brdt_2_SG QuantiTect Primer Assay
4	*BTK*	Rn_Btk_1 FlexiTube siRNA	Rn_Btk_2_SG QuantiTect Primer Assay
5	*Camk2b*	Rn_Camk2b_2 FlexiTube siRNA	Rn_Camk2b_1_SG QuantiTect Primer Assay
6	*Cdk13*	Rn_LOC306998_1 FlexiTube siRNA	Rn_Cdk13_1_SG QuantiTect Primer Assay
7	*Cerk*	Rn_LOC300129_3 FlexiTube siRNA	Rn_Cerk_1_SG QuantiTect Primer Assay
8	*Chka*	Rn_Chka_4 FlexiTube siRNA	Rn_Chka_1_SG QuantiTect Primer Assay
9	*Ckmt1b*	Rn_Ckmt1_1 FlexiTube siRNA	Rn_Ckmt1_2_SG QuantiTect Primer Assay
10	*Clk4*	Rn_LOC287269_2 FlexiTube siRNA	Rn_Clk4_1_SG QuantiTect Primer Assay
11	*Dapk1*	Rn_LOC306722_2 FlexiTube siRNA	Rn_Dapk1_1_SG QuantiTect Primer Assay
12	*Dstyk*	Rn_RGD:735051_3 FlexiTube siRNA	Rn_Dstyk_2_SG QuantiTect Primer Assay
13	*Flt3*	Rn_Flt3_1 FlexiTube siRNA	Rn_Flt3_2_SG QuantiTect Primer Assay
14	*Ibtk*	Rn_LOC315858_2 FlexiTube siRNA	Rn_Ibtk_1_SG QuantiTect Primer Assay
15	*Ilk*	Rn_Ilk_1 FlexiTube siRNA	Rn_RGD:620063_1_SG QuantiTect primer assay
16	*Kit*	Rn_Kit_1 FlexiTube siRNA	Rn_Kit_2_SG QuantiTect Primer Assay
17	*Ksr1*	Rn_LOC360573_3 FlexiTube siRNA	Rn_Ksr1_1_SG QuantiTect Primer Assay
18	*Map4k4*	Rn_LOC301363_4 FlexiTube siRNA	Rn_Map4k4_2_SG QuantiTect Primer Assay
19	*Mapk8ip*	Rn_Mapk8ip_3 FlexiTube siRNA	Rn_Mapk8ip1_1_SG QuantiTect Primer Assay
20	*Mapk8ip3*	Rn_JSAP1_4 FlexiTube siRNA	Rn_Mapk8ip3_2_SG QuantiTect Primer Assay
21	*Mob3c*	Rn_LOC313511_1 FlexiTube siRNA	Rn_Mob3c_1_SG QuantiTect Primer Assay
22	*Pik3ca*	Rn_Pik3ca_4 FlexiTube siRNA	Rn_Pik3ca_2_SG QuantiTect Primer Assay
23	*Prkd1*	Rn_Prkcm_1 FlexiTube siRNA	Rn_Prkd1_1_SG QuantiTect Primer Assay
24	*Prps2*	Rn_Prps2_7 FlexiTube siRNA	Rn_Prps2_1_SG QuantiTect Primer Assay
25	*Srpk1*	Rn_LOC361811_3 FlexiTube siRNA	Rn_Srpk1_1_SG QuantiTect Primer Assay
26	*Tfg*	Rn_LOC360709_2 FlexiTube siRNA	Rn_Tfg_1_SG QuantiTect Primer Assay
27	*Tgfbr1*	Rn_Tgfbr1_3 FlexiTube siRNA	Rn_Tgfbr1_1_SG QuantiTect Primer Assay
28	*Trib3*	Rn_Trib3_5 FlexiTube siRNA	Rn_Trib3_1_SG QuantiTect Primer Assay
29	*Vapa*	Rn_Vapa_2 FlexiTube siRNA	Rn_Vapa_1_SG QuantiTect Primer Assay
30	*Vrk3*	Rn_Vrk3_2 FlexiTube siRNA	Rn_Vrk3_2_SG QuantiTect Primer Assay
31	*Zap70*	Rn_Zap70_1 FlexiTube siRNA	Rn_Zap70_2_SG QuantiTect Primer Assay
32	*Zfp512b*	Rn_LOC311721_2 FlexiTube siRNA	Rn_Zfp512b_2_SG QuantiTect primer assay

Additional file 5. siRNAs identified in the second screening, their target genes, and the corresponding primers for qPCR.
