# Supplementary material for: Identification of kinases and regulatory proteins required for cell migration using a transfected cell-microarray system
Source: BMC Genet. 2015 Feb 5;16:9. doi: 10.1186/s12863-015-0170-7 (PMC4365556; doi:10.1186/s12863-015-0170-7)
Supplement: Additional file 6: — List of cloned genes and their expression vectors. All cDNA fragments were cloned into the multiple cloning sites of pEGFP-N1 or vectors in the pEGFP-C series. [file 12863_2015_170_MOESM6_ESM.pdf]

	Gene name	Species	Expression vector	Cloning sites
1	*Akap12*	rat	pEGFP-N1	EcoRI/SalI
2	*Bmpr1a*	rat	pEGFP-N1	BglII/SalI
3	*Brdt*	rat	pEGFP-C1	BglII/EcoRI
4	*Btk*	human	pEGFP-C1	SalI/SacII
5	*Camk2b*	human	pEGFP-C1	BglII/SalI
6	*Cdk13*	rat	pEGFP-C2	EcoRI/KpnI
7	*Cerk*	rat	pEGFP-N1	EcoRI/SalI
8	*Chka*	rat	pEGFP-C1	SalI/KpnI
9	*Ckmt1b*	rat	pEGFP-N1	BglII/SalI
10	*Clk4*	rat	pEGFP-C2	EcoRI/SalI
11	*Dapk1*	rat	pEGFP-C1	BglII/SalI
12	*Dstyk*	rat	pEGFP-C2	EcoRI/SalI
13	*Flt3*	human	pEGFP-N1	HindIII/PstI
14	*Ibtk*	rat	pEGFP-C1	SalI/KpnI
15	*Ilk*	rat	pEGFP-C1	BglII/SalI
16	*Kit*	rat	pEGFP-N1	EcoRI/SalI
17	*Ksr1*	rat	pEGFP-C2	EcoRI/SalI
18	*Map4k4*	rat	pEGFP-C3	HindIII/EcoRI
19	*Mapk8ip*	rat	pEGFP-C2	EcoRI/SalI
20	*Mapk8ip3*	rat	pEGFP-C2	EcoRI/SalI
21	*Mob3c*	rat	pEGFP-C1	BglII/SalI
22	*Pik3ca*	rat	pEGFP-C1	SalI/KpnI
23	*Prkd1*	human	pEGFP-C1	BglII/EcoRI
24	*Prps2*	rat	pEGFP-C1	BglII/SalI
25	*Srpk1*	rat	pEGFP-C2	EcoRI/SalI
26	*Tfg*	rat	pEGFP-C3	XhoI/SalI
27	*Tgfbr1*	rat	pEGFP-N1	EcoRI/SalI
28	*Trib3*	rat	pEGFP-C2	EcoRI/SalI
29	*Vapa*	rat	pEGFP-C1	BglII/SalI
30	*Vrk3*	rat	pEGFP-C2	EcoRI/SalI
31	*Zap70*	human	pEGFP-N1	HindIII/PstI
32	*Znf512B*	human	pEGFP-N1	HindIII/PstI

Additional file 6. List of cloned genes and their expression vectors.

All cDNA fragments were cloned into the multiple cloning sites of pEGFP-N1 or vectors in the pEGFP-C series.
